# The Hemostasis Apparatus in Pancreatic Cancer and Its Importance beyond Thrombosis

**DOI:** 10.3390/cancers3010267

**Published:** 2011-01-11

**Authors:** Hussein Echrish, Leigh A. Madden, John Greenman, Anthony Maraveyas

**Affiliations:** 1 Centre for Biomedical Research, PGMI, University of Hull, Hull, HU6 7RX, UK; E-Mails: H.H.Echrish@2007.hull.ac.uk (H.E.); L.A.Madden@hull.ac.uk (L.A.M.); J.Greenman@hull.ac.uk (J.G.); 2 Queen's Centre for Oncology and Haematology, Castle Hill Hospital, Castle Road, Cottingham, HU16 5JQ, UK

**Keywords:** pancreatic cancer, thrombosis, hemostasis, tissue factor, microparticles

## Abstract

Laboratory evidence of aberrant coagulation is found in the majority of patients with advanced pancreatic cancer and a clinical consequence of this is the high incidence and prevalence of vascular thromboembolic events. Other sequelae are hypothesized to be the facilitation and acceleration of mechanisms that define the malignant phenotype, such as invasion, trafficking and anchoring, establishing the metastatic niche and inducing angiogenesis. We review the *in vitro* and preclinical evidence that supports the role of the coagulation apparatus in the metastatic process of pancreatic cancer, with a particular emphasis on interaction of this pathway with clinically-targeted growth factor receptor pathways. Links between hemostasis, angiogenesis and epidermal growth factor pathways and their significance as therapeutic targets are considered.

## 1. Introduction

In 1865, Trousseau described thrombosis as a major complication of cancer [1]; the risk of thrombosis being increased by 2–7 fold in patients with cancer compared with a non-cancer population [2-4]. Aberrant coagulation is commonly detected by laboratory tests in many patients with malignancy, especially in advanced or disseminated stages [5]. The higher the prevalence of abnormalities in coagulation the greater the likelihood a recognizable hemostasis-related clinical syndrome will occur. The most commonly observed syndromes in solid malignancies are thromboembolism (TE) and disseminated intravascular coagulation [6].

A seminal post-mortem study reported in 1938 showed that TE is a major complication of pancreatic cancer (PC) with the highest incidence being found in pancreatic tail tumors [7]. Many recent studies have verified that PC is one of the malignancies with the highest prevalence of TE (up to 57% of PC cases) [8-10] and that a patient's risk of developing thrombosis is further increased with chemotherapy treatment [11-14].

The presence of TE in PC patients is correlated with a shorter survival period [15] and the associated early mortality may be directly related to the incidence of TE [16]. There are three, non-mutually exclusive explanations for the relationship between thrombosis in PC and reduced survival. The first is that the thrombosis itself is a potentially lethal event that causes mortality [17] and the use of many of the conventional therapies, including chemotherapy [12,13], anti-angiogenic drugs [18,19] and erythropoietin [20] directly increase this risk. Secondly, malignant cells and the tumor microparticles (MP) are shed into the circulation and are associated with a more malignant phenotype [21], however it remains unclear whether these MP are a direct result, or an epiphenomenon of malignancy. Finally, the aberrant coagulation exacerbates the malignant phenotype by sustaining a continuous loop of factors that promote trafficking and anchoring, invasion, tumor growth and metastasis.

## 2. Tissue Factor in Pancreatic Cancer

**Figure 1.**
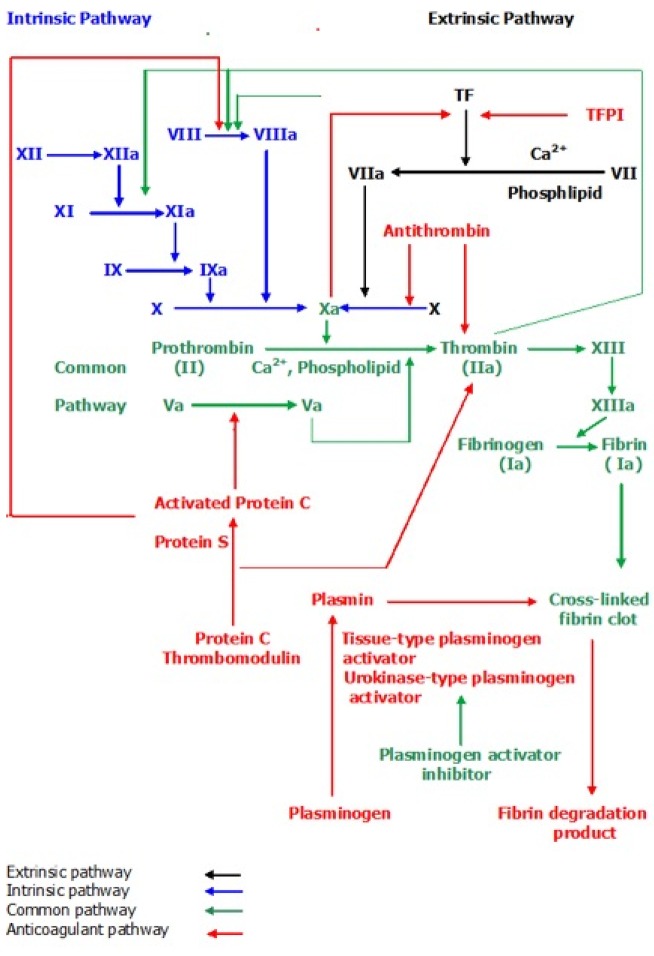
The coagulation cascade.

Tissue factor (TF), designated CD 142, is a glycoprotein receptor [22]. In addition to its principal role in the initiation of the extrinsic pathway of coagulation [23-25] (Figure 1), TF also has a major role in angiogenesis and tumor growth [26-34]. TF consists of three domains (extracellular, transmembrane and cytoplasmic) that have distinct roles [25]. TF is normally expressed in host cells such as endothelial cells, monocytes, macrophages and fibroblasts in response to inflammatory stimuli, or remodeling signals in malignant cases [35]. The plasma of healthy individuals contains relatively minor amounts of biologically active TF [36], but this increases in various conditions including cancer [37]. PC has been associated with increased plasma TF concentration, which is positively correlated with the incidence of TE [11,38].

There are several mechanisms that control TF activity, principally TF pathway inhibitor (TFPI) (Figure 1), which is a serine protease inhibitor [39] released from endothelial cells [40] and acts as an endogenous regulator of TF [41]. TFPI is composed of three Kunitz type protease inhibitor domains and a C-terminal polybasic motif. The first TFPI domain reacts with and inhibits FVII in TF-FVIIa complex; the second TFPI domain reacts with and inhibits FXa in TF-FVII-FXa complex [39,42] and the third domain of TFPI has an unknown function at this time.

**Figure 2.**
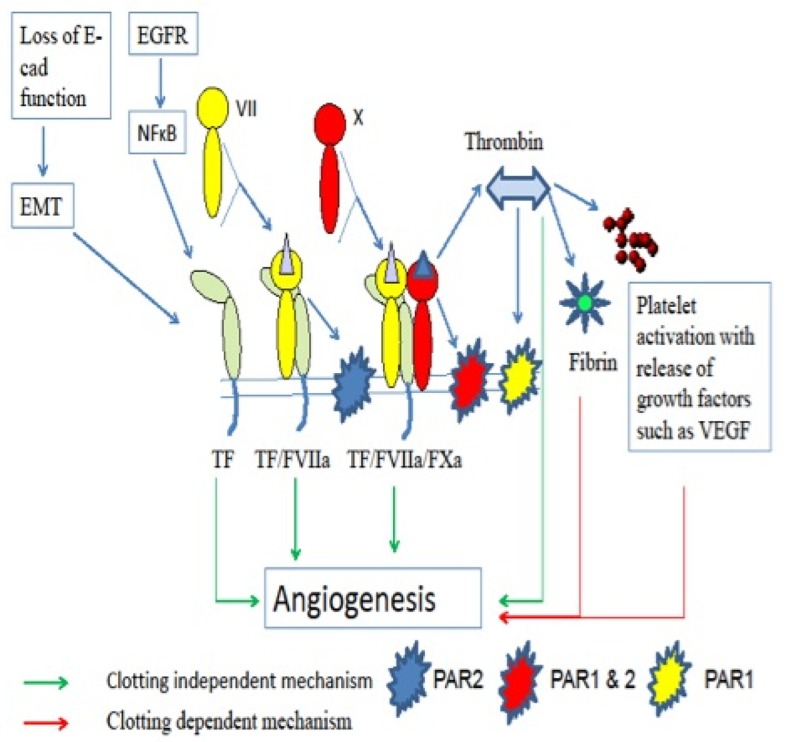
Clotting dependent and independent mechanisms of angiogenesis.

TF expression can be regulated by epidermal growth factor receptor (EGFR) signaling via nuclear factor kappa B (NF-κB) and through the loss of E-cadherin (Figure 2) [43] and by FXa, which has a negative feedback effect on the regulation of TF (Figure 1) [44]. TF expression may also be affected by changes in the activity of phosphatidylinositol 3-kinases (PI3K), and the mitogen-activated protein kinase (MAPK) family members MAPKp38 and extracellular signal-regulated kinases-1/2 (Erk1/2). In tumor cells, the activity of PI3K is decreased and both p38 and Erk1/2 are increased [45] resulting in an increase in cell proliferation.

## 3. Angiogenesis in Pancreatic Cancer

Angiogenesis is defined as new blood vessel formation from the pre-existing vascular network, consisting of three stages: migration, proliferation and differentiation. It can be physiological as in wound healing, tissue remodeling, regeneration and the menstrual cycle or patho-physiological as in cancer, rheumatoid arthritis and atherosclerosis. Tumors can grow up to 1–2 mm^3^ without formation of new blood vessels; however angiogenesis is essential for greater growth [46]. Recently, Lomberk *et al.* suggested that PC is one of the more angiogenesis-driven tumors and rapid tumor growth with a poor prognosis has been positively correlated with increased angiogenesis [47]. There are two mechanisms for promotion of angiogenesis in which proteins of the coagulation pathway can be involved: clotting dependent and clotting independent.

### 3.1. Clotting Dependent Mechanism of Angiogenesis

The extrinsic pathway of the coagulation cascade and clotting dependent mechanism of angiogenesis is initiated by activation of TF receptors via ligand binding; TF binds with FVII to form TF/ FVIIa complex [48]. This complex triggers the coagulation cascade (Figure 1) which involves activation of FX to FXa in the presence of Ca^2+^ and phospholipid (PL), followed by the conversion of prothrombin (FII) to thrombin (FIIa) [48] which is crucial in clot formation due to fibrin formation and platelet activation [49]. Activated platelets can then promote angiogenesis by releasing a number of pro-angiogenic factors such as vascular endothelial factor (VEGF), beta fibroblast growth factor (β-FGF) and platelet derived growth factor (PDGF) [50] (Figure 2).

### 3.2. Clotting Independent Mechanism of Angiogenesis

In addition to its role in the clotting dependent mechanism, thrombin also has a role in the clotting independent mechanism of angiogenesis through proteolytic cleavage of protease-activated receptors (PAR). There are four PAR, of which PAR-1, PAR-3 and PAR-4 are cleaved by thrombin while the proteases trypsin, tryptase, TF-FVIIa and FXa can activate PAR-2 [51-56]. There are several different pathways for clotting independent mechanisms of angiogenesis (Figure 2), including a direct effect of TF-FVIIa that is dependent on phosphorylation of the cytoplasmic domain of TF, as mediated by PAR-2. Other pathways are mediated by FVIIa and FXa activation of PAR-2 [57], for example the TF/FVIIa complex reacts with FXa to form TF/FVIIa/FXa, and this complex triggers protease-activated G protein-coupled receptors, through PAR-1 and PAR-2 [58]. Furthermore, TF-FVIIa-PAR-2 signaling induces the production of pro-angiogenic factors including VEGF [59,60]. In addition, thrombin stimulates adhesion of pancreatic cancer cells to endothelial cells and extracellular matrix [61] and also stimulates gelatinase matrix metalloproteinase-2 (MMP-2), which is a collagen type IV degrading enzyme [62], therefore enhancing invasion of the basement membrane. All these mechanisms contribute to enhance tumor progression, growth, angiogenesis cell invasion.

## 4. Factors That Can Enhance Thrombosis in PC

### 4.1. Extrinsic Factors

The association of increased TE in PC is enhanced with the appearance of distant metastases [63] and conventional superimposed risk factors such as acute medical conditions (e.g., concurrent infection, heart failure and obstructive pulmonary disorders (COPD) [64] and surgery can further exacerbate this risk [65]. Moreover, the systemic treatments used in PC patients may have a significant prothrombotic effect; chemotherapy for example increases the incidence of TE in PC up to 4.8 fold [14]. There are distinct mechanisms which may enhance TE in cancer treated with chemotherapy; platelet activation, decrease of the natural inhibitor factors (such as antithrombin III, protein S and protein C), damage of the blood vessel wall/endothelium [66] and more recently it has been postulated that chemotherapy increases apoptosis in PC [67]. The latter process could lead to apoptotic cells and cellular fragments being increased within the circulation, both having a generally pro-coagulant surface due to TF and PL/phosphatidylserine exposure.

### 4.2. Intrinsic Factors

Tumor cells could directly activate the host cells (endothelial cells, platelets, leucocytes) due to secretion of mucin. Although most of the mucin that enters the blood circulation is cleared by the liver, some of it remains in the circulation and can react with P-selectin on platelets, L-selectin on the leukocytes and P-and E-selectin on vascular endothelium leading to formation of platelet-rich thrombi [68]. Additionally, the expression of many pro-angiogenic factors such as VEGF [69-74], EGFR [73], and platelet-derived endothelial cell growth factor [75] are increased in PC. The expression of VEGF in PC tissue is associated with the microvessel density (MVD) [71]. Furthermore, clinical and laboratory studies suggest that expression of pro-angiogenic markers such as epidermal growth factor (EGF), VEGF, and thymidine phosphorylase on malignant cells including PC are all positively associated with an increase in angiogenesis and a poor prognosis [48,76].

Pro-angiogenic factors enhance tumor cell invasion and angiogenesis as they cause decreased apoptosis, increase survival and cell proliferation [77,78]. Although the mechanism by which pro-angiogenic factors control survival and apoptosis is not clear, it is thought that the binding of these factors results in PI3K/Akt, Ras/MAPK up-regulation [79], and p38MAPK-dependent apoptosis pathways down-regulation [80,82]. Furthermore, VEGF can regulate αvβ3 integrin which enhances cell migration and survival when it is associated with VEGF receptor-2 (VEGFR2) [83,84]. Other contributing mechanisms could include the Ras/MAK, PI3K/Akt, Janus kinase (JAK)/Stat and phospholipase C /protein kinase C pathways which are the main signaling pathways activated by EGFR1, resulting in activation of genes that cause over-expression of angiogenic factors, increased cell proliferation, migration, adhesion, differentiation and apoptosis [85].

## 5. EGFR in Hemostasis and Angiogenesis

It has been noted that over-expression of EGFR1 was seen in 43% of human PC cases and its expression appears to be correlated with poor prognosis, an increased tumor aggressiveness and enhanced angiogenesis [73,86]. EGFR1 expression on PC differs according to the part of the pancreas that is cancerous. For example, cancer of the papilla of Vater did not show an over-expression of EGFR1 compared with a normal control, while cancer of other parts of the pancreas showed over-expression of EGFR1 reaching up to 60%. A range of EGFR2 expression has been reported in PC [87-88] however, no effect on prognosis was observed. The dysregulation of EGFR1 and EGFR2 is involved in oncogenesis [89]. In addition EGFR1 and EGFR2 may affect tumor growth by up-regulation of pro-angiogenic markers such as VEGF [85,90,91], and TF [30] and in this way will enhance angiogenesis and tumor growth. Milson *et al.* postulated that TF expression could be regulated by EGFR, likely controlled partially via NF-κB signaling [43].

Kinase suppressor of Ras1 (KSR1) is involved in the control of both pro-coagulant and aggressive phenotypes of cancer cells by up-regulation of TF downstream of Erb (EGFR) oncogenes [92], and the use of KSR1 targeting agents is being explored as a therapeutic strategy [93-95]. Based on these studies, EGFR-driven up-regulation of TF (and therefore targeting of EGFR receptor directly or indirectly through KSR1) is a potential target for the treatment of PC

## 6. Thrombosis and Microparticles in Pancreatic Cancer

MP are membrane vesicles of approximately 0.1-1 μm in diameter shed from the plasma membrane of healthy, apoptotic and stimulated cells [96,97]. MP express antigenic markers distinctive of the parent cell [98], and may also have a pro-coagulant, negatively charged PL surface, which could support coagulation [99-101]. There is accumulating evidence of a correlation between TF activity expressed on MP and TE in cancers [99]. It has been reported by some investigators that TF bearing MP (TF + MP) involved in coagulation results in an increase of blood clot size rather than initiate coagulation [102], because the TF within the MP is thought to be either at too low a level to trigger clot formation itself or is encrypted; other investigators however, suggest that TF + MP promotes coagulation and has an effect on vascular function [97]. Pro-coagulant activity in cancer patients depends to a large degree on numbers of circulating TF and PL bearing MP [29]. Evidence that the tumor may be the main source of TF + MP comes from the demonstration that TF + MP reduces substantially after successful surgery to remove tumors [101,103] and a correlation between the level of TF + MP activity and number with thrombosis has been demonstrated [21,103-105].

There is cumulative evidence that P-selectin ligand-1 (PSGL-1), in addition to its role in leukocyte trafficking, has a role in blood coagulation due to its effect on TF-containing hybrid MP of platelet and monocyte origin [106,107] which can activate the coagulation cascade and enhance the TF-FVIIa complex proteolytic activity [108]. For this reason P-selectin has been used as an independent indicator for TE risk [109].

It has also been demonstrated that tumour growth, metastasis, angiogenesis and thrombosis can be stimulated by TF + MP [29,31-34] . Furthermore, increased levels of MP can be found in patients with a number of cancers but the highest levels of TF activity are those in PC patients [99,104]. In a series of 37 cancer-free controls, 23 advanced PC and 17 advanced breast cancer, the upper limit of TF activity in the normal range was 273 fM Xa min^-1^. Thirty-four percent of PC patients and 29% of advanced breast cancer cases involved in this study had TF activity above the upper limit of the normal range. Interestingly, there was no correlation between the absolute number of MP and the reported TF activity [103].

Tesselaar *et al.* [104] used the same upper limit for normal range of TF activity, and also reported that the TF activity associated with TF + MP was significantly higher in cancer patients than non-cancer patients. For 51 cancer patients with thrombosis the mean TF activity was 1125 fM Xa min^−1^ (19–12,333 fM Xa min^-1^). This group consisted of patients with the following types of tumor: 14 colorectal, 10 pancreatic, six testicular, four renal, three ovarian, two esophageal, two prostatic, two bone, two laryngeal, two breast, two respiratory tract, one bile duct and one adrenal cancer. Of these, the highest noted MP associated TF activity was 2,080 fM Xa min^−1^ (510–12,333) in PC. TF activity was also relatively high (55–1,578 fM Xa min^−1^) in colorectal cancer. The range of other tumors studied showed a TF activity between 80–603 fM Xa min^−1^ [104]. The mean TF + MP activity in 49 cancer patients without thrombosis was 162 fM Xa min^−1^ (23–535 fM Xa min^−1^). In a series of nine patients with lung cancer, three with breast cancer, one with PC, one with renal, one with sarcoma and 23 healthy controls, Tilley *et al.* reported that PC has the highest TF activity (48.3 pM) of all malignancies tested within the study [99].

Zwicker *et al.* observed that there was no statistical difference in TF + MP numbers in healthy controls and non-small cell lung cancer, while TF + MP were significantly higher in pancreatic cancer (32 of 47 PC cases) [105]. It was reported that the highest incidence of patients with TF + MP above a lower detectable limit was observed in PC (25 of 39) followed by colorectal carcinoma (7 of 12), compared with non-cancer controls (6 of 31) and that the number of TF + MP in PC and colorectal cancer was significantly higher than in non-cancer controls [101]. However, the difference in the number of TF + MP compared with non-cancer controls was insignificant in lung cancer (5 of 28), breast cancer (4 of 9) and ovarian cancer (5 of 8) suggesting that TF + MP have an important role in the pro-coagulant phenotype and may be a contributing factor related to the high incidence of TE associated with PC patients [29].

## 7. The Clinical Experience of Targeting Growth Factor Receptors in Pancreatic Cancer

From the clinical perspective, two recently completed trials of antiangiogenic agents (bevacizumab or Avastin®) and axitinib (S1005 and AG-013736)], when used as a first line treatment in combination with standard therapy in the setting of PC, yielded disappointing results. Firstly, axitnib in combination with gemcitabine resulted in a median overall survival of 7.4 months, compared to 8.2 months for gemcitabine alone, in 630 patients [110]. Secondly, bevacizumab, in combination with gemcitabine also failed to confer any increase in overall survival (5.8 months) when compared to gemcitabine alone (5.9 months) in a study of 535 patients [111]. Gemcitabine is one of the standard chemotherapy treatments that has been used widely in PC and has a cytotoxic action toward proliferative cells during the S phase of the cell cycle [112]. Furthermore, in a large (607 patients) randomized phase III clinical trial, where a triple combination of bevacizumab , erlotinib and gemcitabine was compared to gemcitabine, erlotinib and placebo, no statistical difference in the median overall survival rate was found (7.1 *vs.* 6.0 months respectively; p = 0.209) [113]. A concern remains that these agents also drive thrombosis and whatever potential benefit from anti-cancer treatments may exist may be lost through excess thrombotic events [17].

Two other recently concluded trials of anti-EGFR agents [erlotinib (Tarceva®) and cetuximab (Erbitux)] in the setting of advanced pancreatic cancer (APC) have yielded conflicting results. A large randomized clinical trial of Tarceva® and gemcitabine *vs.* gemcitabine alone produced one of the few positive results in APC [14]. In this series of 569 cases of APC, the median overall survival was significantly prolonged with Tarceva® (100 mg/ daily) and gemcitabine compared with gemcitabine only (6.24 *vs.* 5.91 months, p = 0.038); a marginal clinical improvement that was seen only in the stage IV patients. There was no significant difference in the objective response rate and there were many grade I and II side effects in the group treated with Tarceva® and gemcitabine, such as rash, diarrhea, stomatitis and infection [14]. However, a larger randomized clinical trial of cetuximab (IgG1 monoclonal antibody against EGFR) and gemcitabine *vs.* gemcitabine alone was negative. In this randomized controlled trial of 745 patients with APC, the gemcitabine and cetuximab did not demonstrate a survival prolongation compared to gemcitabine alone (6.3 months for gemcitabine plus cetuximab *vs.* 5.9 months for gemcitabine alone; p = 0.23) [114]. To date, therefore, there is little positive outcome demonstrated for the targeting of growth factor receptors in PC.

## 8. Clinical Evidence of Benefit from Direct Interference with the Coagulation Apparatus

Heparin can potentially act as an anti-cancer factor via a variety of mechanisms. It inhibits several coagulation factors such as thrombin, FXa, FIXa, FXIa, FXIIa [50] and inhibits cell proliferation, particularly of those cells that over express PAR-1 [115]. Heparin also it interacts with VEGF-165 and VEGF-189 expressed on malignant cells [116] and could inhibit angiogenesis via blocking of P-and L-selectin [117]. We have recently shown that low molecular weight heparin (LMWH) can decrease the angiogenic and chemoattractant activity of PC patients' sera [118].

A recent randomized phase IIb study in APC of gemcitabine *vs.* weight-adjusted dalteparin, a type of LMWH, demonstrated a significant decrease in the overall incidence of venous TE from 31% to 12%, with a reduction of recorded lethal TE and sudden death from 9% to 0%. These differences were significant but the trial was too small to demonstrate an overall survival advantage [119]. In a second larger randomized trial, Reiss *et al.* [120] studied 312 APC patients receiving chemotherapy randomized into two groups: those with enoxaparin (Fragmin®), which is another type of LMWH, and those without enoxaparin. Similar to Maraveyas *et al.*, a significant decrease of clinical TE was found 5% *vs.* 15%, respectively, in the group treated with LMWH. However, once again no overall survival benefit was documented [120]. Epstein *et al.* [121] studied 6,870 PC cases of which 19% of patients suffered TE, 95% of PC patients that developed TE were treated with chemotherapy and LMWH and the survival time of PC patients who developed thrombosis at the time of the diagnosis was 6.2 months while that of those with secondary thrombosis after PC was 13.7 months [120].

## 9. Conclusions

There is a clinical correlation between short survival time related to TE in PC; however, the underlying drivers of this phenomenon are complex. The use of LMWH can decrease plasma levels of TF, reduce thrombin activation and reduce the incidence of TE. Whether there is an overall survival gain from these treatments as suggested by some meta-analyses for mixed cancer populations [122] is dependent upon future, appropriately powered studies. There is significant experimental evidence that the coagulation pathway and the growth factor pathways are linked exerting significant influence on the cancer process. However, randomized controlled clinical studies of single agent targeting of these pathways in conjunction with chemotherapy have not provided evidence of clinical benefit to date. More recently even, a dual targeting approach (EGFR1 and VEGF inhibition) has not produced survival benefit.

It is possible that refinement of these targeting strategies will also need the concurrent use of heparin-based treatment. Potentially beneficial impact could result through enhancement of the ‘targeted’ effects while simultaneously offsetting any prothrombotic impact these targeted molecules may have.
